# Toe flexor strength is associated with mobility in older adults with pronated and supinated feet but not with neutral feet

**DOI:** 10.1186/s13047-020-00422-y

**Published:** 2020-09-11

**Authors:** Yuki Kusagawa, Toshiyuki Kurihara, Aiko Imai, Sumiaki Maeo, Takashi Sugiyama, Hiroaki Kanehisa, Tadao Isaka

**Affiliations:** 1 Graduate School of Sport and Health Science, Ritsumeikan University, 1-1-1 Noji Higashi, Kusatsu, Shiga 525-8577 Japan; 2 Research Organization of Science and Technology, Ritsumeikan University, Kusatsu, Shiga Japan; 3 Department of Rehabilitation and Care, Faculty of Health Sciences, Suzuka University of Medical Sciences, Suzuka, Mie Japan; 4 Faculty of Care and Rehabilitation, Seijoh University, Tokai, Nagoya, Japan; 5 Ritsumeikan Global Innovation Research Organization, Ritsumeikan University, Kusatsu, Shiga Japan; 6 Faculty of Sport and Health Science, Ritsumeikan University, Kusatsu, Shiga Japan

**Keywords:** Foot alignment, Foot posture index, Toe grip strength, Functional performance, Comfortable walking speed, Ageing

## Abstract

**Background:**

Older adults are known to have more pronated foot posture and decreased toe flexor strength (TFS), as well as decreased mobility in daily life compared to young adults. Although foot posture is reported to be an influential factor for walking biomechanics in young adults, there is less information on this subject in older adults. Age-related reduction in TFS is shown to be associated with impairments of functional performance, but it is poorly understood whether foot posture influences the relationships between TFS and functional performances. Therefore, the present study aimed to elucidate this concern by examining older women.

**Methods:**

Seventy community-dwelling older women (76.8 ± 4.4 years) voluntarily participated in this study. Foot posture was evaluated by the 6-item foot posture index (FPI). Based on the FPI score, participants were allocated to pronated, neutral, or supinated group (*n* = 33, 26, and 11, respectively). TFS was assessed using a toe grip dynamometer in a seated position. Scores of 30-s chair stand, timed up-and-go, 5-m comfortable-speed walking, and static balance tests were determined to evaluate functional performances. Pearson’s correlation coefficients were computed to examine the relationships between TFS and functional performances in each group.

**Results:**

TFS positively correlated with comfortable walking speed in the pronated (*r* = 0.37, *p* = 0.03) and supinated (*r* = 0.76, *p* < 0.001) groups, but not in the neutral group (*r* = 0.17, *p* = 0.42). For the two significant relationships, an analysis of covariance showed that there was no significant difference between the pronated and supinated groups in the slopes of the regression lines, suggesting a similar relative contribution of TFS to comfortable walking speed between the two groups. In addition, TFS tended to negatively correlate with timed up-and-go time in the pronated (*r* = − 0.32, *p* = 0.07) and supinated (*r* = − 0.56, *p* = 0.08) groups, and positively correlate with 30-s chair stand score in the pronated group (*r* = 0.31, *p* = 0.08).

**Conclusions:**

The present study indicates that TFS would be associated with mobility, walking performance in particular, in older women with pronated and supinated feet but not with neutral feet.

## Background

A human foot has a wide variation of postures ranging from a pronated to supinated foot [1]. Compared to young and middle-aged adults, older adults tend to have a more pronated foot [1] and lowered medial longitudinal arch [2]. Thus, the changes in foot posture towards a more pronated position are recognized as a part of the ageing process [1]. In addition, it is known that a pronated foot and supinated foot have a higher occurrence of lower extremity injuries (e.g. medial tibial stress syndrome [3], patellofemoral pain [3], and stress fracture of lower limb and foot [4]). Moreover, flatfoot deformity with posterior tibial tendon dysfunction, characterized by an overpronated foot, is associated with decreased mobility in daily life (hereafter simply referred to as “mobility”), such as slower walking speed, shorter stride length, and decreased cadence compared to asymptomatic adults [5].

Foot posture may influence functional performances because pronated and supinated feet are known to altered the biomechanics on the lower extremity compared to neutral feet [6, 7]. Previous studies on walking biomechanics for young and middle-aged adults have shown that pronated and supinated feet alter the rearfoot frontal plane motion compared to neutral feet [6, 7], plantar pressure distribution [8], and the muscle activities of lower limbs [9]. Older adults with a higher occurrence of a pronated foot demonstrate greater medial displacement of the center of pressure [10] and smaller midfoot and metatarsal range of motion on the sagittal plane, as well as less plantarflexed calcaneus at toe-off, which may collectivity explain their less propulsive gait pattern [11]. However, the previous studies have failed to find a close relationship between foot posture and mobility in older adults. For example, foot posture based on the scores of the 6-item foot posture index (FPI) had a significant but poor correlation (*r* = − 0.176) with walking speed in older adults [12]. While, it has been shown that the score of the timed up-and-go test did not differ among different foot posture groups [13]. These findings imply that not foot posture per se but other factors influence mobility in older adults.

It is known that the function of the first metatarsal joint, which exerts toe flexor strength (TFS), is vital for maintaining postural balance during walking [14]. The TFS decreases by approximately 30% in the 70s compared to 20s [15], and this may be accompanied by decreased functional performances. For example, weakness in TFS in older adults evaluated by a toe grip dynamometer is related with decreased walking speed and stride length [16], as well as longer timed up-and-go test time and decreased functional reach [17]. Moreover, TFS determined by using a force plate is shown to be strongly correlated with the anterior limit of the functional base of support [18]. In addition, reduced TFS is considered to be a risk factor for falls in older adults [19, 20]. These findings indicate that TFS can be a determinant factor for mobility in older adults. However, there is less information on how foot posture influences the relationships between TFS and functional performances in older adults.

To our knowledge, only one study investigated inter-relationships among foot posture, TFS, and functional performance. Menz et al. [12] showed that while older adults with insufficient TFS had decreased mobility (assessed by walking speed, the score of chair stand, and postural balance) compared to older adults with sufficient TFS, FPI score was not selected as an independent predictor of balance and functional performance. This suggests that for older adults, foot posture does not influence relationships between TFS and functional performances. Referring to the prior findings, however, it should be pointed out that TFS was evaluated by using a paper grip test, being a non-quantitative measurement. This test would be useful in clinical practice [21], but it appears methodologically weak for examining the potential relationships of TFS with other quantitative variables. In contrast to the paper grip test, TFS can be quantitatively evaluated by a toe grip dynamometer [15, 16, 22–25], and this method has good reliability for detecting TFS in older adults [26]. Thus, the previous findings on the inter-relationships among foot posture, TFS, and functional performances in older adults should be reexamined through the determination of TFS with a toe grip dynamometer. The present study determined TFS by using a quantitative method and aimed to elucidate how TFS is associated with functional performances in relation to foot posture in older women.

## Methods

### Participants

Seventy community-dwelling healthy women aged over 65 years old (age, 76.8 ± 4.4 yrs.; height, 150.1 ± 5.0 cm; body weight, 51.8 ± 5.9 kg; mean ± standard deviation [SD]) voluntarily participated in this study. Participants were excluded if they had any history of a diagnosed neuromuscular disorder or lower limb injury. All participants were free of ambulation disability, functionally independent in daily living, and none of the participants used canes or other walking aids. This study was approved by The Research Ethics Committee of Seijoh University (15OT23) and that of Ritsumeikan University (BKC-2018-084). All participants provided prior written informed consent based on the guidelines of the Declaration of Helsinki.

### Measurements

#### Foot posture

Foot posture was evaluated by the 6-item foot posture index (FPI) [1]. First, participants were asked to stand in a relaxed stance position with double limb support, looking straight ahead. Participants maintained this position and one examiner (YK) assessed their right foot by six clinical criteria in accordance with the procedure described in a prior study [1]: talar head palpation, supra and infra lateral malleolar curvature, calcaneal frontal plane position, prominence in the region of the talonavicular joint, congruence of the medial longitudinal arch, and abduction/adduction of the forefoot on the rearfoot. A digital camera placed approximately 25 cm from the posterior aspect of the calcaneus was used to record the calcaneal frontal plane position. The calcaneal inversion/eversion angle was measured from the images using ImageJ software. The score of each criterion ranged from − 2 to + 2, and the scores of the six items were summed. On the basis of the total score, the participants were allocated to one of three groups: pronated (+ 6 to + 12), neutral (0 to + 5), or supinated (− 1 to − 12), according to previous study [13]. For the evaluation of the FPI, the examiner had excellent intra-rater between-day reliability (intraclass correlation coefficient = 0.92). Normalized truncated navicular height (NTNH) in the double limb standing position was also calculated as the ratio of navicular height relative to the truncated foot length [27]. Truncated foot length and navicular height were determined as the distance from the first metatarsophalangeal joint to the most posterior aspect of the heel and from the most medial prominence point of the navicular tuberosity to the floor, respectively.

#### Toe flexor strength (TFS)

TFS of the right foot was measured using a commercially available isometric dynamometer (T.K.K. 3361, Takei Scientific Instrument Co., Niigata), in accordance with the procedure adopted in a previous study [22]. Participants generated TFS in a seated position with their hip and knee joints flexed at 90 degrees and the ankle joint at a neutral position. TFS has been measured in this (seated) position by many studies [15, 16, 22–25] and shown to be correlated with the cross-sectional area of toe flexors [22] and functional performances [16, 17]. The participants placed their right foot on the dynamometer, adjusted their posterior heel position at the heel stopper, and gripped their toes at the grip-bar. During the measurements, the participants were instructed to cross their arms in front of their chest and perform the task without any extraneous movements. Before the measurements, the participants generated submaximal forces 2–3 times to familiarize themselves with the measurement procedure. After the completion of the familiarization trials and a rest period of 3 min, the participants performed the task with maximal effort for at least 3 s. The maximal trial was repeated twice with at least one-minute rest, and the larger value of the two measurements was used for further analysis. The intraclass correlation coefficient for the two measurements was 0.90 in this study. The unit of body weight was converted from “kilogram (kg)” to “ kilogram-weight (kgw)” in order to match the dimension of units for TFS (N) and body weight, and the TFS was expressed as the value relative to body weight (N/kgw).

#### Timed up-and-go test time

The timed up-and-go test, which assesses agility and dynamic balance, was conducted using a procedure reported by Rikli and Jones [28]. Prior to the measurement, the participants were fully seated on a chair with their back against the chair at the height of 45 cm. Then they stood up and walked a distance of 3-m walkway as quickly as possible without running, turned around, and then walked back to the chair and sat down with the back against the chair again. The time taken from standing up from the chair to sitting down on the chair was determined using a stopwatch. The test was repeated twice with at least one-minute rest and the lower (faster) value was adopted for further analysis.

#### 5-m comfortable walking speed

To evaluate walking ability, the present study conducted a 5-m comfortable-speed walking test in accordance with a procedure used in a prior study [29]. The participants walked at their usual pace on a straight and flat 11-m walkway on the indoor surface. The time over a 5 m distance between tape marks set at 3 and 8 m from the start of the walkway was measured using a stopwatch. Walking speed (m/s) was calculated by dividing the distance by the time. The test was repeated twice with at least one-minute rest and the faster value was used for further analysis.

#### Scores of thirty-second chair stand

The 30-s chair stand test, which assesses lower body strength, was performed using a 45-cm high chair without an armrest as described in a prior study [28]. The participants were asked to start from the seated position and stand up with their legs straight and then sit down with full weight on the chair. During the measurements, the participants repeated the chair stand task as many and quickly as possible within 30 s, with their arms crossed over the chest. The number of completed chair stand task in 30 s was adopted as the score. Two trials were performed with at least one-minute rest and the better score was used for further analysis.

#### Static balance

Center of pressure (COP) trajectories were determined with eyes-opened (EO) and eyes-closed (EC) conditions, both in the double-leg stance, to evaluate static balance [30]. In the EO condition, participants stood barefoot quietly for 10 s in the middle of a plantar pressure measurement platform (FDS, zebris Medical GmbH, Germany) while keeping their hands on their waist and gazing forward at the mark on the wall 2 m away at the level of their eyes. The COP trajectories were measured at a sampling rate of 120 Hz. The participants then performed the trial in the EC condition with their eyes closed in the same posture as above for 10 s. Postural sway velocity was calculated by dividing the COP trajectories by the trial time (10 s) in both conditions. The Romberg ratio (EC COP trajectories/ EO COP trajectories) was also analyzed according to a previous study [30].

#### Statistical analysis

Descriptive data are presented as means ± SDs. ﻿The normality of the measured variables was assessed using the Shapiro-Wilk test, and all variables except for postural sway velocity at the EC condition and Romberg’s ratio were confirmed as normally distributed. Thus, the postural sway velocity at the EC condition and Romberg’s ratio were log transformed and the all subsequent analysis was used parametric tests. One-way analysis of variance (ANOVA) followed by a Bonferroni test was used to analyze the differences among the three groups on the measured variables. Pearson’s correlation coefficients were computed to examine the relationships between TFS and functional performances in each group. When significant correlations were found in multiple groups within the same variable, an analysis of covariance (ANCOVA) was used to test the effect of foot posture on the slopes of the regression lines. The level of significance was set at *p* < 0.05. All data were analyzed using statistical software (SPSS 26.0, IBM Co., USA).

## Results

The number of the participants allocated to each group was 33, 26, and 11 for the pronated, neutral, and supinated groups, respectively. The FPI score of each group was 8.1 ± 1.8 for the pronated, 3.0 ± 1.6 for the neutral, and − 3.0 ± 1.6 for the supinated group.

Table 1 summarizes the descriptive data on the physical and foot characteristics of each group. There were no significant differences among the groups in all variables (*p* = 0.36–0.74) except for NTNH and navicular height. The NTNH was significantly lower in the pronated group than in the neutral and supinated groups (*p* < 0.001), and also lower in the neutral group than in the supinated group (*p* < 0.001). The navicular height was significantly lower in the pronated group than in the neutral (*p* < 0.001) and supinated (*p* < 0.001) groups.

**Table 1:** Physical and foot characteristics of each of the pronated, neutral, and supinated groups

	Pronated group (*n* = 33)	Neutral group (*n* = 26)	Supinated group (*n* = 11)
Age (years)	76.7 ± 4.8	78.0 ± 3.2	74.5 ± 5.0
Body height (cm)	150.7 ± 5.7	149.2 ± 2.9	150.4 ± 7.1
Body weight (kg)	51.8 ± 6.5	52.7 ± 5.7	49.4 ± 4.1
BMI (kg/m^2^)	22.9 ± 3.0	23.7 ± 2.8	21.9 ± 2.2
Truncated foot length (cm)	16.8 ± 0.8	16.9 ± 0.7	16.7 ± 0.9
Navicular height (cm)	3.8 ± 0.4 ^*†^	4.1 ± 0.4	4.4 ± 0.3 ^*^
NTNH	0.22 ± 0.03 ^*†^	0.24 ± 0.02	0.26 ± 0.02 ^*^

Values are means ± SDs

*BMI* Body mass index, *NTNH* Normalized truncated navicular height

*Significantly different from the neutral group (*p* < 0.001)

†Significantly different from the supinated group (*p* < 0.001)

Table 2 shows descriptive data on TFS and functional performances. None of them differed among the three groups (*p* = 0.58–0.83).

**Table 2:** Descriptive data on toe flexor strength and functional performance in each of the pronated, neutral, and supinated groups

	Pronated group (*n* = 33)	Neutral group (*n* = 26)	Supinated group (*n* = 11)
Toe flexor strength (N/kgw)	0.10 ± 0.05	0.10 ± 0.04	0.09 ± 0.04
Scores of thirty-second chair stand (times/30s)	23.4 ± 4.7	23.5 ± 4.9	23.3 ± 7.3
Comfortable walking speed (m/s)	1.43 ± 0.23	1.39 ± 0.19	1.39 ± 0.29
Timed Up-and-Go test time (s)	5.72 ± 0.87	5.81 ± 0.79	5.84 ± 1.96
Postural sway velocity at the EO condition (cm/s)	0.67 ± 0.26	0.71 ± 0.30	0.63 ± 0.30
Postural sway velocity at the EC condition (cm/s)	1.06 ± 0.58	0.98 ± 0.38	1.26 ± 1.05
Romberg’s ratio (EC/EO)	1.65 ± 0.81	1.51 ± 0.60	1.88 ± 0.74

Values are means ± SDs

*EO* Eyes-opened, *EC* Eyes-closed

**Fig. 1:**
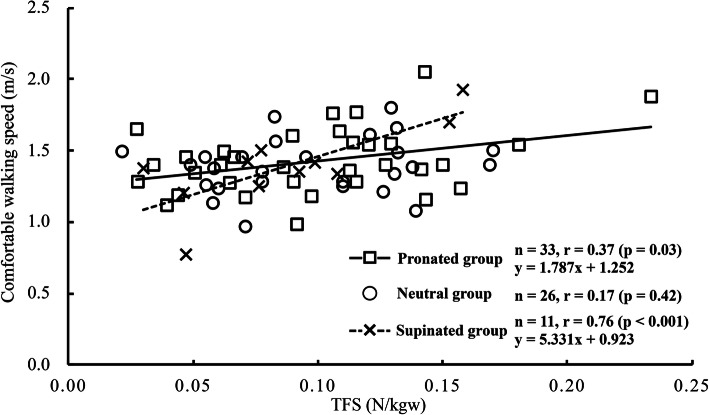
Relationship between TFS and comfortable walking speed in each of the pronated, neutral, and supinated groups

Significant positive correlations were observed between TFS and comfortable walking speed in the pronated (*r* = 0.37, *p* = 0.03) and supinated (*r* = 0.76, *p* < 0.001) groups (Fig. 1), but not in the neutral group (*r* = 0.17, *p* = 0.42). For the two significant relationships, ANCOVA showed that the slope of the regression line did not differ between the pronated and supinated groups (*p* = 0.60). TFS trended to negatively correlate with the timed up-and-go test time in the pronated (*r* = − 0.32, *p* = 0.07) and supinated (*r* = − 0.56, *p* = 0.08) groups, and positively correlate with the score of chair stand in the pronated group (*r* = 0.31, *p* = 0.08). The other functional performances were not significantly correlated with TFS in any groups (*r* = − 0.46 − 0.31, *p* = 0.16–0.97).

## Discussion

A major finding obtained here was that TFS was significantly correlated with comfortable walking speed in the pronated and supinated groups. In addition, TFS tended to negatively correlate with the timed up-and-go in the pronated and supinated groups and positively correlate with the score of chair stand in the pronated group. The corresponding relationships were not found in the neutral group. These results indicate that TFS is associated with mobility, walking speed in particular, in older women with pronated and supinated feet but not with neutral feet.

The current study showed that foot posture influences the relationship between TFS and functional performances in older women, while a previous study suggested that foot posture does not influence functional performances in older adults [12]. This may be due to the differences between the present and prior studies in the procedure for the TFS measurement and analytical approach to examining the association of TFS with functional performances. First, the previous study evaluated TFS by a paper grip test [12], which is not quantitative. On the other hand, the present study quantitatively determined TFS by using a toe grip dynamometer and examined the relationships with the quantitative variables. Second, the previous study adopted not only TFS and functional performance but also FPI as parametric variables and examined the relationships between them [12]. The current study used FPI as a categorical variable rather than a parametric variable and examined the relationship between TFS and functional performance in each group based on the classification of FPI. These differences in the methodological and analytical approaches may explain the discrepancy between the present and previous studies in the results of the influences of foot posture on the association of TFS with functional performance.

For the observed significant relationships in the pronated and supinated groups, ANCOVA showed no significant difference between the two groups in the slopes of the regression lines. This implies that the relative contribution of TFS on comfortable walking speed does not differ between the pronated and supinated groups. The reason why the significant associations were found in older women with pronated and supinated feet is unknown but might partially involve the possible influences of foot posture on the biomechanical profiles of a foot during the gait cycle.

First, pronated feet compared to neutral feet demonstrate greater rearfoot inversion angle [31], and higher activity of tibialis anterior and lower activity of peroneus longus [9] at the initial contact of the gait cycle. At the midstance of the gait cycle, young adults with pronated feet have an increased rearfoot eversion angle [31], and increased activity of the tibialis posterior and decreased activity of the peroneus longus [9]. In the stance phase of the gait cycle, furthermore, pronated feet would require more prolonged activity of intrinsic muscles to stabilize the transverse tarsal joint, which contributes to propulsive force generation, compared to neutral feet [32]. Impairment of functions of the intrinsic foot muscles by the nerve block [33] and smaller size of these muscles [34] are reported to be associated with foot pronation. The cross-sectional area of the intrinsic foot muscles is a determinant factor for TFS [22]. Thus, the intrinsic foot muscles appear to have an important role in developing TFS [22] and for maintaining the medial longitudinal arch, contributing to the support of foot posture [33, 34]. Taking these aspects into account together with the aging effects on TFS [15], older adults with pronated feet may be required to activate the intrinsic foot muscles to a greater degree, which directly leads to greater TFS development, to compensate for the aforementioned abnormal joint kinematics and muscle activities of the lower limbs during walking. Consequently, this might partially explain the significant association between TFS and comfortable walking speed.

As compared to individuals with pronated feet, however, those with supinated feet have a decreased peak rearfoot eversion angle and midfoot eversion angle during walking [7]. In addition, supinated feet show greater peak plantar pressure at the 2nd, 3rd, and 4th metatarsal head and smaller peak plantar pressure at the hallux during walking, compared to neutral feet [35]. These findings suggest that walking biomechanics in supinated feet would differ from that of either pronated or neutral feet. At the same time, it implies that the aforementioned reason assumed for the significant association between TFS and comfortable walking speed in the pronated group cannot be applied to the supinated foot group. However, this somewhat differs from the assumption derived from the ANCOVA results indicating a similar contribution of TFS development to comfortable walking speed between pronated and supinated foot groups. In any case, there is less information on biomechanical profiles during the gait cycle in individuals with supinated feet. Further study is needed to clarify this concern.

In contrast to the pronated and supinated foot groups, the neutral foot group did not show a significant relationship between TFS and comfortable walking speed. Individuals with neutral feet do not have abnormal walking biomechanics, observed in those with pronated and supinated feet. In other words, possessing high TFS may not be an advantage for individuals with neutral feet to walk faster, as there is no abnormal walking biomechanics to compensate for. Considering these aspects, it seems that for older adults with neutral foot posture, TFS does not make a substantial contribution to propelling the body in the forward direction during walking.

In the current results, there was no significant relationship between TFS and static balance regardless of the groups. For older adults, no study has examined the association of TFS, determined quantitatively using a dynamometer, with postural static balance. For older adults, many physiological factors are associated with postural balance [36], such as weakness of hip strength and knee extensor strength [37]. From a systematic review, however, evidence indicating the contribution of muscle weakness to postural instability in healthy older adults is limited [38]. Combining this with the current results, it is likely that muscle strength, including TFS, may not be an influential factor for postural balance regardless of the difference of the foot posture.

The present study has some limitations. First, this study examined only older women. There are no previous studies examining only women to investigate the relationship between TFS and functional performances. Moreover, it is known that the age-related reduction in TFS is different between men and women [15]. Thus, whether the current findings can be applied to older men is yet to be investigated. Second, the present study did not measure body kinematics and activities of the related muscles during the execution of the functional tasks. Future studies should be directed towards including measurements of these biomechanical parameters to elucidate the physiological backgrounds of the influences of foot posture on the relationship between TFS and mobility in older adults. Third, the current study did not measure the force generation capacity of other muscles located around the hip, knee, and ankle joints. As a general observation, hip extension [39] and hip abduction [40] strength are associated with comfortable walking speed in older adults. In addition, it has been shown that older adults produce net positive work more at the hip joint than at the ankle joint during walking [41]. Therefore, further study is warranted to determine the contribution of TFS to functional performances in combination with other lower limb muscles.

## Conclusion

In older women, TFS was positively correlated with comfortable walking speed in the pronated and supinated groups, but not in the neutral group. The results of the present study suggest that for podiatrists and other medical staff, the quantitative evaluation of TFS and careful assessment of foot posture would be useful for improving mobility in older women. Notably, the findings obtained here indicate that strengthening TFS might be beneficial for older women with pronated/supinated feet to increase mobility, such as walking speed.

## Data Availability

Please contact the corresponding author for data request.

## Abbreviations

EC: Eyes closed
EO: Eyes opened
COP: Center of pressure
FPI: Foot posture index
NTNH: Normalized truncated navicular height
TFS: Toe flexor strength
